# AI-support for the detection of intracranial large vessel occlusions: One-year prospective evaluation

**DOI:** 10.1016/j.heliyon.2023.e19065

**Published:** 2023-08-10

**Authors:** K.G. van Leeuwen, M.J. Becks, D. Grob, F. de Lange, J.H.E. Rutten, S. Schalekamp, M.J.C.M. Rutten, B. van Ginneken, M. de Rooij, F.J.A. Meijer

**Affiliations:** a Department of Medical Imaging, Radboud University Medical Center, Nijmegen, the Netherlands; b Department of Radiology, Jeroen Bosch Hospital, ‘s-Hertogenbosch, the Netherlands

**Keywords:** Stroke, Artificial intelligence, Cerebrovascular occlusion, Evaluation study, Computed tomography angiography

## Abstract

**Purpose:**

Few studies have evaluated real-world performance of radiological AI-tools in clinical practice. Over one-year, we prospectively evaluated the use of AI software to support the detection of intracranial large vessel occlusions (LVO) on CT angiography (CTA).

**Method:**

Quantitative measures (user log-in attempts, AI standalone performance) and qualitative data (user surveys) were reviewed by a key-user group at three timepoints. A total of 491 CTA studies of 460 patients were included for analysis.

**Results:**

The overall accuracy of the AI-tool for LVO detection and localization was 87.6%, sensitivity 69.1% and specificity 91.2%. Out of 81 LVOs, 31 of 34 (91%) M1 occlusions were detected correctly, 19 of 38 (50%) M2 occlusions, and 6 of 9 (67%) ICA occlusions. The product was considered user-friendly. The diagnostic confidence of the users for LVO detection remained the same over the year. The last measured net promotor score was −56%. The use of the AI-tool fluctuated over the year with a declining trend.

**Conclusions:**

Our pragmatic approach of evaluating the AI-tool used in clinical practice, helped us to monitor the usage, to estimate the perceived added value by the users of the AI-tool, and to make an informed decision about the continuation of the use of the AI-tool.

## Highlights

• Prospective monitoring of AI-tools in clinical practice informs about added value.
• The value of an AI-tool depends on the diagnostic performance for its intended use, the clinical context, and the expectations of the end-users.
• Our evaluation suggested that our primary goals to increase diagnostic performance and confidence of the users to detect large vessel occlusions were not met.

## Nomenclature

**Abbreviations**
AI: Artificial Intelligence
CTA: CT angiography
LVO: large vessel occlusion
ICA: internal carotid artery
NPS: Net Promotor Score

## 1 Introduction

In the last decade, there is an incremental workload for radiologists in a clinical environment where specialized expertise is demanded. In stroke centers, a 24/7 neuroradiology service is needed for a fast and accurate diagnostic work-up of patients presenting with an acute neurological deficit. This is necessary for treatment decision making with narrow treatment windows, in order to improve the clinical outcome of the patient [[1], [2], [3]]. Nowadays, artificial intelligence (AI)-tools are available to support the workflow and radiologists in making a quick and accurate diagnosis [[4], [5], [6], [7]]. Clinically relevant abnormalities can be subtle and overlooked, especially in a busy first aid department, during night shifts, or in case of less experienced readers (e.g., residents). However, few studies are available that have evaluated the actual diagnostic performance and added value of AI-tools applied in routine clinical practice [6,[8], [9], [10]].

The evaluation of AI tools in routine clinical practice is relevant for several reasons. The first reason is patient safety. Even though products are cleared by e.g. the FDA (US) or a Notified Body (Europe), this does not mean they have been validated in a real-world environment. Interaction with existing systems and human-AI interactions may provide different results than retrospective validation [11,12]. Secondly, better informed purchase decisions can be made when understanding the value of the product [13]. Thirdly, it is relevant for post market surveillance. Medical device manufacturers are obliged to gather data from clinical practice (e.g. efficacy, feedback, adverse events) to ensure safety and enable product improvements. As they usually do not have direct access to the clinical data or feedback, it is a shared responsibility by the clinical users and the vendor to monitor safe use and effectiveness of the software [12,14]. Lastly, to have AI products reimbursed, in most countries, it is necessary to demonstrate the positive clinical impact, for which prospective evaluation is key [9].

In our academic stroke center, we implemented an AI-tool with the aim to improve the diagnostic performance and confidence of (resident) radiologists for the detection of intracranial large vessel occlusions (LVO) on CT angiography (CTA). To monitor the impact of the tool and decide on the continued use after the pilot, we evaluated the AI-tool with a protocol leading to minimal administration burden and technical efforts.

In this article, we share the methods and results of our one-year prospective clinical evaluation.

## 2 Materials and methods

### 2.1 Clinical implementation of the AI-tool

An AI-tool to support brain CT analysis (StrokeViewer v3, Nicolab, Amsterdam, the Netherlands) was implemented at our radiology department for a trial period of one year (January 2021–December 2021). StrokeViewer includes AI-tools for LVO detection and localization, intracranial hemorrhage detection, collateral assessment, perfusion CT analysis, and enables data sharing between stroke hub and spoke centers with remote access to radiological studies via a browser or phone application.

At our department, CT studies in the diagnostic work-up of acute cerebral stroke are evaluated by radiologists and residents with variable experience in stroke imaging. All CTA images were automatically sent to StrokeViewer for analysis. The radiologists and residents were prompted with an email when the AI-results were available and could choose to access the results in a web browser application. Our center opted not to enable automated push-to-PACS of the AI-results. The mobile app notification functionality was not yet available when the tool was implemented and was not adopted when it came available halfway the evaluation period. In the first month, user trainings were provided.

### 2.2 Population

**Fig. 1:**
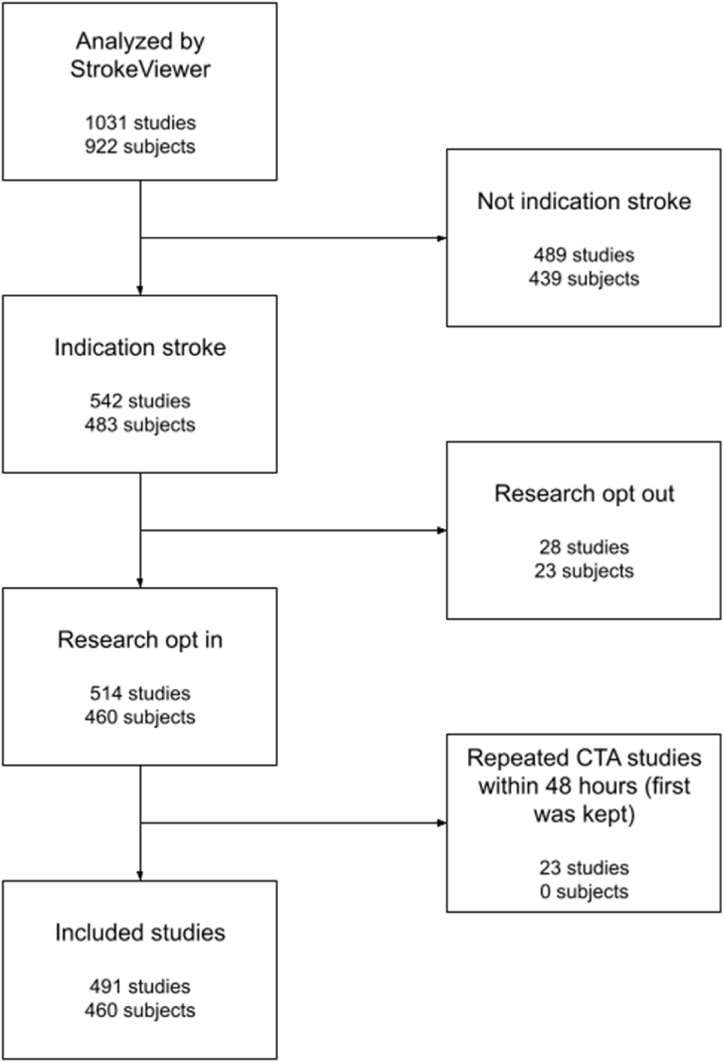
Flowchart of data included in the evaluation.

The AI-tool analyzed 1031 brain CT studies of 922 patients, of which 542 studies of 483 patients were acquired for the suspicion of acute ischemic stroke. Studies were excluded from patients who did not consent to research use of their data at time of hospital admission (23 subjects) or patients who had repeated CTA examinations within two days (first was kept, 24 studies). This resulted in a set of 491 studies of 460 patients as shown in Fig. 1. The evaluation study was approved by the institutional review board and informed consent was waived. Data was anonymized before being used for analysis.

### 2.3 Evaluation methods

The evaluation methodology was based on the plan-do-study-act cycle (Supplemental Materials A) [15]. The metrics were specified according to a framework presented in the NHS handbook [15]. The use of the software (log-in attempts, number of unique users, used product features), diagnostic confidence (subjective confidence level), and diagnostic performance (stand-alone accuracy, sensitivity, specificity) were obtained (details in Supplemental Materials B).

The reference standard was set by an experienced neuroradiologist having access to initial diagnosis, the AI-tool results, and clinical (follow-up) information. Occlusion detection of occlusions in the first (M1) and second (M2) segment of the middle cerebral artery and in the internal carotid artery (ICA) were part of the intended use of the AI-tool. All other occlusion types were considered negative in the reference standard (n = 31). Analysis was performed on a per-lesion basis; occlusions detected in the wrong location by the AI-tool were considered missed (false negative).

Log-in attempts were provided by the vendor (timestamp with an anonymized user ID). Multiple log-in attempts within an hour by the same user were considered a single login attempt, as to avoid duplicate counts for users logging in multiple times to check if the results are already available. Surveys (Supplemental Materials C) were sent out to all users at three time points. All data were analyzed and reviewed by the key-user group at three timepoints (Supplemental Materials D).

## 3 Results

*Diagnostic accuracy of the AI-tool.* The stand-alone diagnostic accuracy of the AI-tool for LVO detection and localization was 87.6%, with a sensitivity of 69.1% and specificity 91.2%. Out of 81 LVOs (M1 = 34, M2 = 38, ICA = 9), 25 were not detected by the AI-tool: 20 were missed (M2 = 15, M1 = 2, ICA = 3) and 5 were detected at an incorrect location (M2 = 4, M1 = 1). This resulted in a sensitivity of 91% for M1, 50% for M2, and 67% for ICA occlusions. Table 1 shows the confusion matrix.

**Table 1:** Confusion matrix occlusion detection of M1, M2 and ICA.

Reference → AI tool ↓	LVO	No LVO
LVO	56 (TP)	36 (FP)
No LVO	25 (FN)^a^	374 (TN)
	Sensitivity = 69.1%	Specificity = 91.2%

LVO = large vessel occlusion, ICA = internal carotid artery, TP = True Positives, FP = False Positives, FN = False Negatives, TN = True Negatives.

a FN occlusions included 5 occlusions detected in the wrong location (M2 = 4, M1 = 1) and 20 occlusions missed (M2 = 15, M1 = 2, ICA = 3).

### 3.1 Usage and survey results

**Fig. 2:**
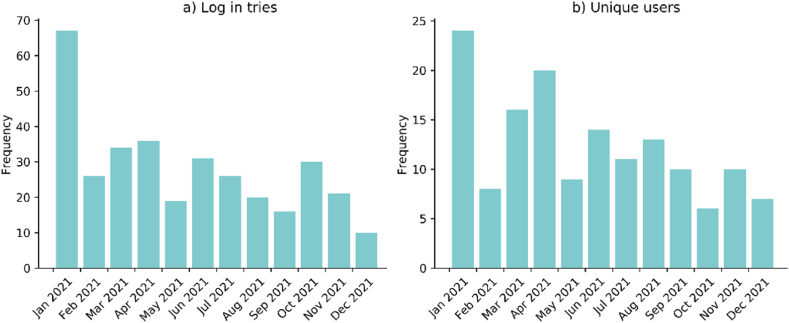
AI-tool use (radiologists and residents) per month. Figure a) shows the login attempts and b) the unique users. Multiple login attempts within 1 h by a single user, where considered as a single login.

Throughout the year, the clinical team with access to the results varied due to staffing changes, but typically included around 40 radiologists and residents. In total, 54 unique users logged in to the AI-tool. The number of monthly unique users and login attempts fluctuated, with a declining trend, as shown in Fig. 2. The first month included training of the users. The self-reported use of the AI-tool for LVO diagnostics varied over the respondents. Eight respondents reported ‘never’ or ‘rarely’, three ‘sometimes’, and six answered ‘often’ or ‘always’.

The three surveys disseminated throughout the pilot year yielded a response of 19, 22 and 17 users, representing a mix of residents (10, 10, 6), neuroradiologists (2, 3, 2), and non-neuroradiologists (7, 9, 9). Table 2 shows the main results. Self-reported confidence for LVO diagnostics remained stable. The AI-tool was considered user-friendly (average of 7.4/10). The product was not likely to be missed in the future if the implementation would be halted, but the score slightly improved in the last survey (2.9/10 Feb, 2.8/10 Jun, 3.8/10 Dec). The question ‘How likely would you be to recommend the tool to colleagues?’ was posed to calculate the Net Promotor Score (NPS). This is calculated by the percentage of promotors (scoring a 9 or 10) minus the percentage of detractors (scoring a 6 or lower). The NPS was −64% in Feb, −70% in Jun and −56% in Dec showing the users were not particularly satisfied with the AI-tool. Scores provided by residents and (neuro)radiologists were similar. A subanalysis of the scores is provided in Supplemental Materials E. When asked if the user wanted the AI-software to stay available after the one-year trial period, 7 responded ‘Yes’, 5 responded ‘No’, 3 were neutral and 2 considered keeping it for other purposes other than diagnostics (research and image sharing). A selection of free text elaborations can be found in Supplemental Materials F.

**Table 2:** Main survey outcomes.

Question	Feb 2021 (n = 14/19)^a^	Jun 2021 (n = 20/22)^a^	Dec 2021 (n = 16/17)^a^
**How confident do you feel at diagnosing vessel occlusions?** Mean of score between 1 and 10	7.9	7.9	7.9
**How user friendly do you consider the tool?** Mean of score between 1 and 10	8.1	7.0	7.2
**If the tool wouldn't be there anymore, how much would you miss it?** Mean of score between 1 and 10	2.9	2.8	3.8
**How likely would you be to recommend the tool to colleagues?** Net Promoter Score = %promotors-%detractors. Scale from -100 to 100.	−64%	−70%	−56%

Feb = February, Jun = June, Dec = December.

a Respondents that answered to have never used the AI tool were excluded from the analysis.

## 4 Discussion

The usage metrics and survey results suggest that users experienced limited added value of the applied AI-tool in clinical practice. One of the reasons could be that the diagnostic performance of the LVO detection and localization algorithm alone may have not been sufficient to meet expectations in our clinical context. The performance of the LVO detection and localization algorithm was similar to previous studies (sensitivity 72%–77% and specificity 78%–88%) with a slightly lower sensitivity (69%) and higher specificity (91%) at our center [16,17]. Most of the missed LVOs by the AI-tool included the M2 vessels, which are also more difficult to detect by radiologists. Most other commercially available AI-tools currently on the market do not (yet) detect M2 occlusions [18,19]. Another reason the users may have experienced limited added value is because of their (unconscious) misinterpretation of the intended use. The AI-tool only detects LVOs in the anterior circulation, but no posterior vessel occlusions. If the user would have false expectations of the intended use to detect all intracranial vessel occlusions, one may experience an unjustified low sensitivity of the AI-tool of 51%.

It is important to acknowledge that this AI-tool provides more functionalities than LVO localization. We have used and evaluated only a part of the product in this study. Furthermore, the push-to-PACS functionality of the AI-results was not activated and the radiologists needed to actively retrieve the AI-results. This additional step may have contributed to the decline in the usage of the AI-tool.

The AI for radiology industry is still maturing and clinical efficacy of the tools is not always evident [10]. Therefore, the monitoring of an AI-tool after implementation may help to evaluate whether the primary goals are met and whether continuation of the use is prudent. It may not always be feasible, nor necessary, to perform an extensive clinical study. Our method of evaluation, using data that was easy to collect with little administration burden, helped us to critically review the performance, usage and the value of the AI-tool implemented in clinical practice.

A limitation, inherent to the design of our evaluation study, is that we do not know how the AI-tool has influenced the diagnostic accuracy of the radiologists for LVO detection. Our study was more qualitative and exploratory by design. A previous study reported that radiologists perceived no added value of the computer aided detection software, while it actually improved their diagnostic performance [20]. We know from an early health-technology assessment that when the number of missed treatable occlusions is reduced by the AI-tool, it can be a cost-effective solution [21]. In a different center, the AI-tool retrospectively detected LVOs that were clinically missed [16], demonstrating potential added value. However, estimates of the percentage of occlusions missed by radiologists range between 6% and 20%, which remains far less than the percentage of missed occlusions by the AI-tool (30.9%) [22].

## 5 Conclusion

The primary goal of implementing the AI-tool at our department was to improve the performance and confidence of the (resident) radiologists for LVO detection. Considering the limited sensitivity of the AI-tool for LVO detection and localization, its fluctuating use, the survey results, and the software price point with lack of reimbursement in our healthcare system, our department decided to discontinue the use of the AI-tool for LVO detection. Our department however still believes in the potential added value of AI for LVO detection and may reconsider when available products have matured and have demonstrated improved diagnostic performance, especially for the detection of M2 occlusions. Furthermore, it would be of added value to also aid in the detection of vessel occlusions in the posterior circulation.

## Funding

The authors state that this work has not received any funding.

## Production notes

### Author contribution statement

Kicky G van Leeuwen and Frederick JA Meijer: Conceived and designed the experiments, Performed the experiments; Analyzed and interpreted the data; Wrote the paper.

Marinus J Becks, Dagmar Grob, Frank de Lange, Johan HE Rutten and Maarten de Rooij: conceived and designed the experiments, performed the experiments and analyzed and interpreted the data.

Steven Schalekamp, Matthieu JCM Rutten and Bram van Ginneken: analyzed and interpreted the data.

### Data availability statement

Data included in article/supplementary material/referenced in article.

## Declaration of competing interest

The authors declare that they have no known competing financial interests or personal relationships that could have appeared to influence the work reported in this paper.

## Appendix A Supplementary data

Appendix A Evaluation cycle. Appendix A

Appendix B Tables with metric definitions. Appendix B

Appendix C Survey questions. Appendix C

Appendix D Data collection and evaluation flowchart. Appendix D

Appendix E Survey results of residents vs. radiologists. Appendix E
