# Supplementary material for: AI-support for the detection of intracranial large vessel occlusions: One-year prospective evaluation
Source: Heliyon. 2023 Aug 10;9(8):e19065. doi: 10.1016/j.heliyon.2023.e19065 (PMC10458691; doi:10.1016/j.heliyon.2023.e19065)

A. Evaluation cycle

Based on thevideo from Mike Davidge, Head of Measurement at the NHS Institute for Innovation and Improvement. https://www.youtube.com/watch?v=Za1o77jAnbw. Permission was granted for reuse.

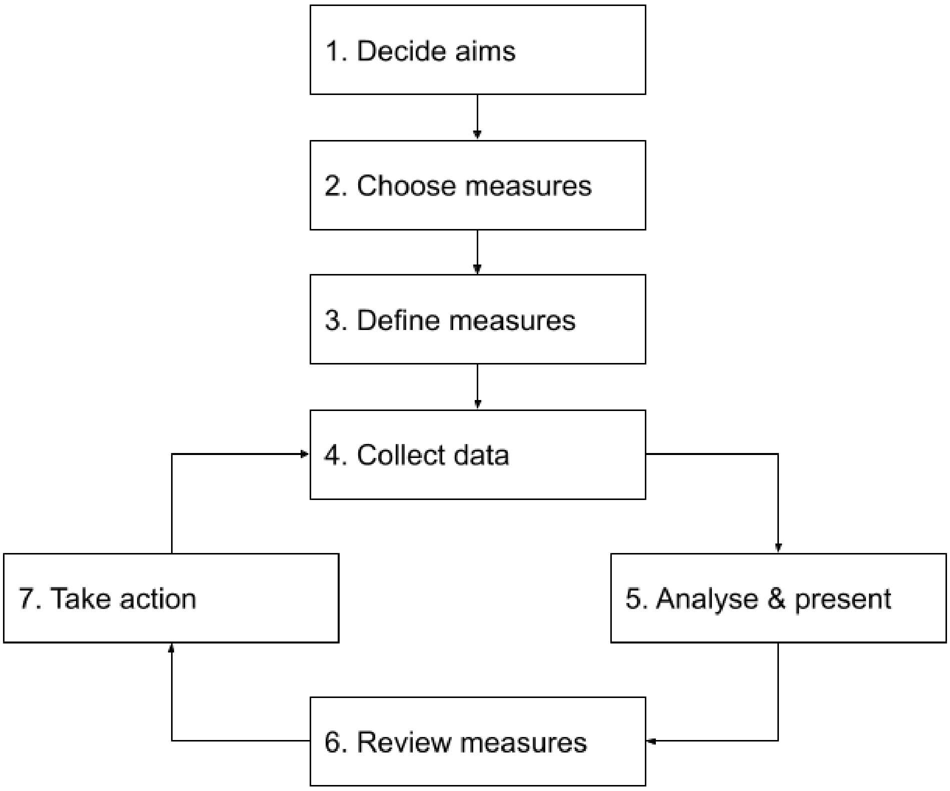

Supplement: Appendix A — Evaluation cycle. [file mmc1.pdf]
