# Supplementary material for: AI-support for the detection of intracranial large vessel occlusions: One-year prospective evaluation
Source: Heliyon. 2023 Aug 10;9(8):e19065. doi: 10.1016/j.heliyon.2023.e19065 (PMC10458691; doi:10.1016/j.heliyon.2023.e19065)
Supplement: Appendix B — Tables with metric definitions. [file mmc2.pdf]

B. Tables with metric definitions

The table format used has been based on The NHS Handbook of Quality and Service Improvement Tools, chapter 5.3 by the NHS Institute for Innovation and Improvement.

**Title**<br>Metric summary	**Clinical use**
**Purpose**<br>Consider the purpose of measuring this aspect of performance. If there is no good reason, do you really need to measure it?	Define the adoption of the software. Measure if the software is being used.
**Target**<br>Which performance targets should you set and by when? This communicates precisely what you are trying to achieve.	- Number of unique users per month<br>- Number of login attempts per month
**Formula**<br>How do you calculate the performance measure? Be precise: the formula must include exactly what you are measuring to avoid any confusion.	- Exclude user that reviewed the cases to set the reference standard<br>- Multiple login attempts in the same hour were considered a single login attempt.
**Frequency**<br>Decide how often you will measure and how often you will review the measure itself.	According to the evaluation timeline.
**Who measures?**<br>Identify the person responsible for the measure.	Evaluation lead
**Source of data**<br>Specify the source of data so you can use the measure consistently. This way you can compare performance between periods accurately.	StrokeViewer logs (anonymized), shared by vendor.
**Who takes action?**<br>Who is responsible for taking action on the measure?	Initiated by evaluation lead, discussed with the clinical lead.
**What do they do?**<br>Specify the types of action people should take to improve the performance of the measure.	Understand why people use/do not use the product and intervene accordingly (e.g. discuss in team meeting, notify about existence, extra training).

**Title**<br>Metric summary	**Diagnostic performance**
**Purpose**<br>Consider the purpose of measuring this aspect of performance. If there is no good reason, do you really need to measure it?	Get an indication of the added value of the tool on clinical practice → reduced missed vessel occlusions
**Target**	Accuracy occlusion based

Which performance targets should you set and by when? This communicates precisely what you are trying to achieve.	Sensitivity<br>Specificity
**Formula**<br>How do you calculate the performance measure? Be precise: the formula must include exactly what you are measuring to avoid any confusion.	Accuracy = (TP+TN)/total<br>Sens = TP/(TP+FN)<br>Spec = TN/(TN+FP)<br><br>Reference = retrospective expert second read with clinical follow-up information.<br>Wrong location considered missed.<br>Occlusions other than M1, M2, ICA considered negative.
**Frequency**<br>Decide how often you will measure and how often you will review the measure itself.	According to the evaluation timeline.
**Who measures?**<br>Identify the person responsible for the measure.	Clinical team
**Source of data**<br>Specify the source of data so you can use the measure consistently. This way you can compare performance between periods accurately.	Reference standard: PACS, EHR, StrokeViewer web application.<br><br>Stand-alone software: StrokeViewer web application
**Who takes action?**<br>Who is responsible for taking action on the measure?	The project team.
**What do they do?**<br>Specify the types of action people should take to improve the performance of the measure.	Discuss results with the vendor regularly.<br>Determine whether interventions are necessary/possible.<br>Determine whether or not to continue with the use of the product.

**Title**<br>Metric summary	**Confidence in LVO diagnostics**
**Purpose**<br>Consider the purpose of measuring this aspect of performance. If there is no good reason, do you really need to measure it?	Measure the impact of the software on the radiologists' and residents' confidence for diagnosing LVOs.
**Target**<br>Which performance targets should you set and by when? This communicates precisely what you are trying to achieve.	Change in self-reported confidence over time.
**Formula**<br>How do you calculate the performance measure? Be precise: the formula must include exactly what you are measuring to avoid any confusion.	How confident do you feel at diagnosing intracerebral vessel occlusions? Rated between 1 (very insecure) and 10 (very confident)
**Frequency**<br>Decide how often you will measure and how often you will review the measure itself.	According to the evaluation timeline. Review at the end of the pilot.

**Who measures?**<br>Identify the person responsible for the measure.	Evaluation lead.
**Source of data**<br>Specify the source of data so you can use the measure consistently. This way you can compare performance between periods accurately.	Survey provided to all users.
**Who takes what action?**<br>Specify the types of action people should take to improve the performance of the measure.	Determine whether or not to continue with the use of the product. By the project team.
