# Supplementary material for: AI-support for the detection of intracranial large vessel occlusions: One-year prospective evaluation
Source: Heliyon. 2023 Aug 10;9(8):e19065. doi: 10.1016/j.heliyon.2023.e19065 (PMC10458691; doi:10.1016/j.heliyon.2023.e19065)
Supplement: Appendix C — Survey questions. [file mmc3.pdf]

C. Survey questions

Translated from Dutch to English, as provided in the last survey round.

Question	Type	Answer options
Name	Short answer	
Role	Dropdown	Resident; Fellow-neuro; Non-neuro radiologist; Neuroradiologist
How often do you use StrokeViewerfor<br>- Vessel occlusion diagnostics<br>- ICH diagnostics<br>- Remote image viewing<br>- Collateral assessment	Multiple-choice grid	Never; Rarely; Sometimes; Often; Always
How confident do you feel at diagnosing intracerebral vessel occlusions?	Linear scale	1 (very insecure) to 10 (very confident)
When assessing a scan from patients with indication stroke, how often do you ask advice from a colleague?	Percentage	0-100%
How user friendly is StrokeViewer?	Linear scale	1 (not user friendly) to 10 (very user friendly)
If StrokeViewer wouldn't be there anymore, how much would you miss it?	Linear scale, paragraph	1 (not at all) to 10 (I can no longer do without)
How likely would you be to recommend StrokeViewer to a colleague?	Linear scale, paragraph	1 (not likely) to 10 (very likely)
Would you like StrokeViewer to remain after the trial period?	Multiple choice	Yes; No; Other, ...
Other questions or comments	Paragraph	
