# Supplementary figures and images for: AI-support for the detection of intracranial large vessel occlusions: One-year prospective evaluation
Source: Heliyon. 2023 Aug 10;9(8):e19065. doi: 10.1016/j.heliyon.2023.e19065 (PMC10458691; doi:10.1016/j.heliyon.2023.e19065)

## D. Data collection and evaluation flowchart

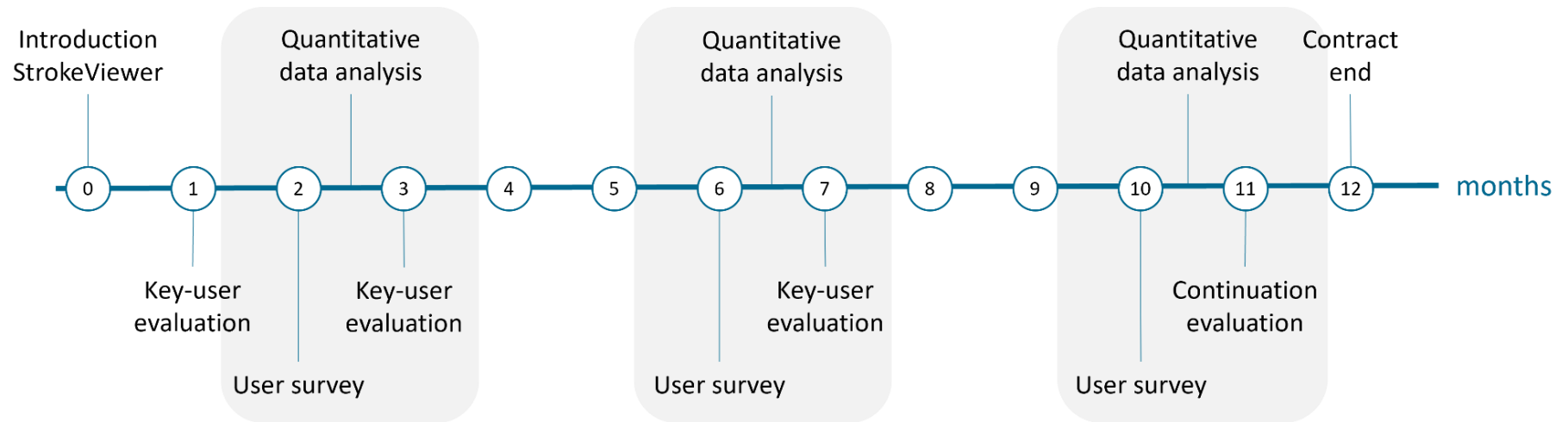

Supplement: Appendix D — Data collection and evaluation flowchart. [file mmc4.pdf]
