# Supplementary material for: AI-support for the detection of intracranial large vessel occlusions: One-year prospective evaluation
Source: Heliyon. 2023 Aug 10;9(8):e19065. doi: 10.1016/j.heliyon.2023.e19065 (PMC10458691; doi:10.1016/j.heliyon.2023.e19065)
Supplement: Appendix E — Survey results of residents vs. radiologists. [file mmc5.pdf]

E. Survey results of residents vs. radiologists

Main survey outcomes as presented in Table 2, splitting the residents (Res) from the (neuro)radiologists (Rad)

**Question**	**Feb 2021** (n=14/19)*		**Jun 2021** (n=20/22)*		**Dec 2021** (n=16/17)*	
	Res (n=6)	Rad (n=8)	Res (n=9)	Rad (n=11)	Res (n=6)	Rad (n=10)
**How confident do you feel at diagnosing vessel occlusions?** *Mean of score between 1 and 10*	7.3	8.2	7.7	8.1	7.5	8.1
**How user friendly do you consider the tool?** *Mean of score between 1 and 10*	7.8	8.1	7.7	6.5	7.0	7.4
**If the tool wouldn't be there anymore, how much would you miss it?** *Mean of score between 1 and 10*	3.2	2.5	2.9	2.6	4.7	3.2
**How likely would you be to recommend the tool to colleagues?** *Net Promoter Score = %promotors- %detractors. Scale from -100 to 100.*	-67%	-62%	-67%	-72%	-50%	-60%

**Respondents that answered to have never used the AI tool were excluded from the analysis.*
Res = residents, Rad = (neuro)radiologists.
Feb = February, Jun = June, Dec = December
